# Supplementary material for: Control of a programmed cell death pathway in Pseudomonas aeruginosa by an antiterminator
Source: Nat Commun. 2021 Mar 17;12:1702. doi: 10.1038/s41467-021-21941-7 (PMC7969949; doi:10.1038/s41467-021-21941-7)
Supplement: Supplementary file 1 — Supplementary Information [file 41467_2021_21941_MOESM1_ESM.pdf]

SUPPLEMENTARY INFORMATION

Control of a programmed cell death pathway in *Pseudomonas aeruginosa* by an antiterminator

Jennifer M. Peña[1], Samantha M. Prezioso[1], Kirsty A. McFarland[1], Tracy K. Kambara[1],
Kathryn M. Ramsey[1,2#], Padraig Deighan[3], and Simon L. Dove[1]*

1. Division of Infectious Diseases, Boston Children's Hospital, Harvard Medical School, Boston, Massachusetts 02115, USA.
2. Departments of Cell and Molecular Biology and Biomedical and Pharmaceutical Sciences, University of Rhode Island, Kingston, Rhode Island, USA.
3. Department of Biology, Emmanuel College, Boston, Massachusetts 02115, USA.

These authors contributed equally: Samantha M. Prezioso and Kirsty A. McFarland

[#]Current address: Departments of Cell and Molecular Biology and Biomedical and Pharmaceutical Sciences, University of Rhode Island, Kingston, Rhode Island, USA.

*To whom correspondence may be addressed. Email: simon.dove@childrens.harvard.edu

CONTENTS

Supplementary Figures 1-10.

Supplementary Tables 1-5.

Supplementary References.

SUPPLEMENTARY FIGURES

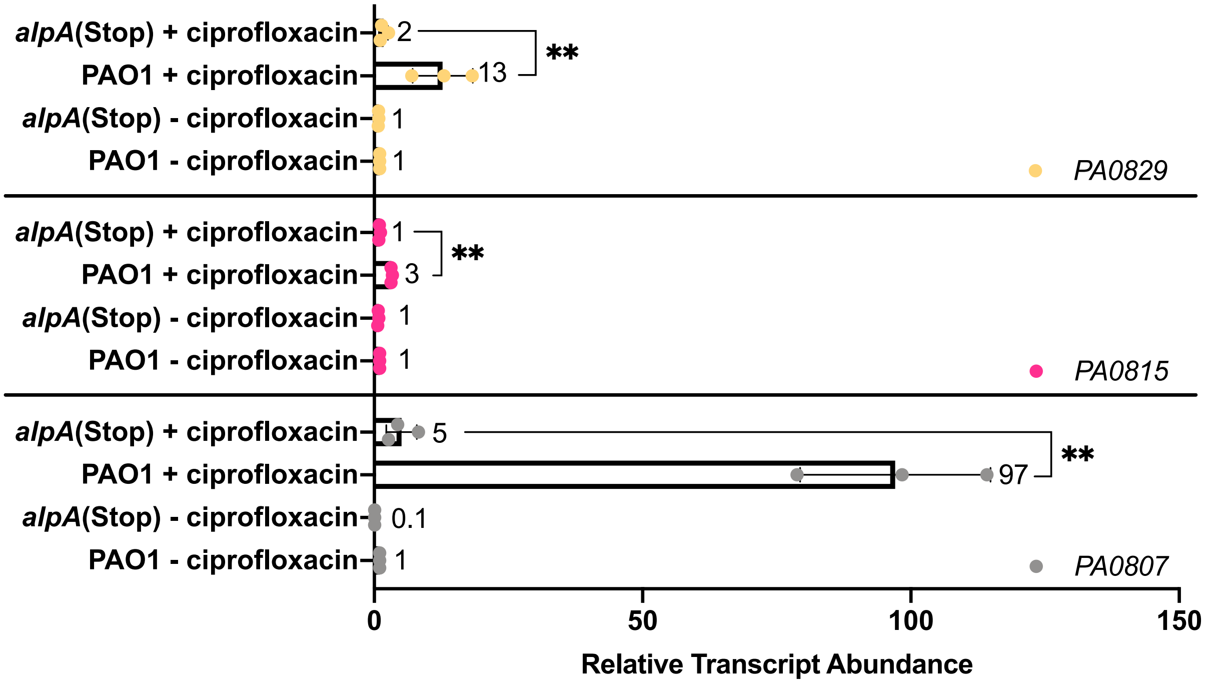

Supplementary Fig. 1. **AlpA positively regulates genes encoded on the positive strand from *PA0807* to *PA0829***. Transcript abundance of the indicated genes was measured using qRT-PCR in PAO1 WT and *alpA*(Stop) mutant cells treated with and without ciprofloxacin. Values and error bars reflect mean ± SD of n=3 biological replicates. 2-tailed, unpaired, unequal variance t-tests were used to calculate p-values between PAO1 strains treated with ciprofloxacin and *alpA*(Stop) strains treated with ciprofloxacin. p-values indicated by the following symbols: ≤0.01 = **.

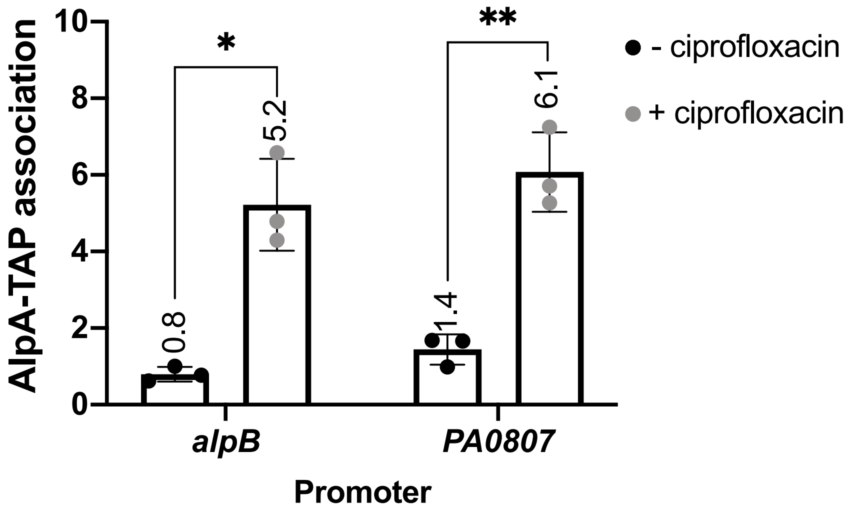

Supplementary Fig. 2. AlpA associates with the *alpB* and *PA0807* promoter regions. Chromatin immunoprecipitation (ChIP) of AlpA-TAP in cells treated with ciprofloxacin or untreated. Enrichment of indicated genomic regions following ChIP with AlpA-TAP as determined by qPCR. Values and error bars reflect mean ± SD of n=3 biological replicates. 2-tailed, unpaired, unequal variance t-tests were used to calculate p-values between indicated samples. p-values indicated by the following symbols: ≤0.05 = *, ≤0.01 = **.

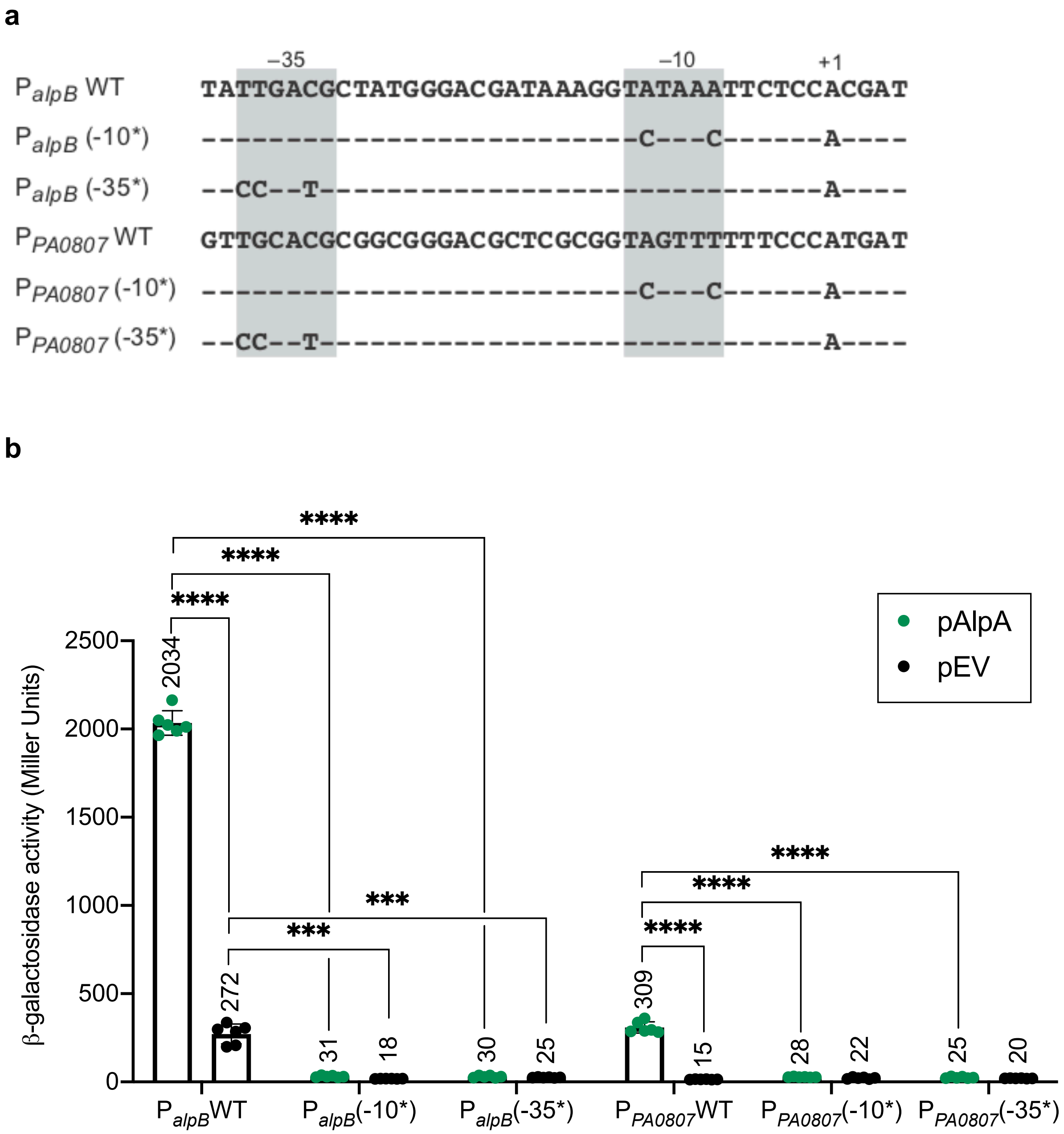

Supplementary Fig. 3. Mutations in the -35 and -10 elements of the predicted *alpB* and *PA0807* promoters. a Diagram of point mutations made in the putative -35 and -10 elements of the *alpB* and *PA0807* promoters. **b** β-galactosidase activity (in Miller Units) was measured from the indicated reporter constructs in PAO1. Cells contained plasmid pAlpA (green) or plasmid pEV (the empty vector; black). Decrease in *lacZ* expression from the mutated promoter constructs confirms the location of the -35 and -10 elements and establishes the promoter sequences for the *alpB* and *PA0807* operons. Values and error bars reflect mean ± SD of n=3 biological replicates in technical duplicate. 2-tailed, unpaired, unequal variance t-tests were used to calculate p-values between indicated samples. p-values indicated by the following symbols: ≤0.001 = ***, ≤0.0001 = ****.

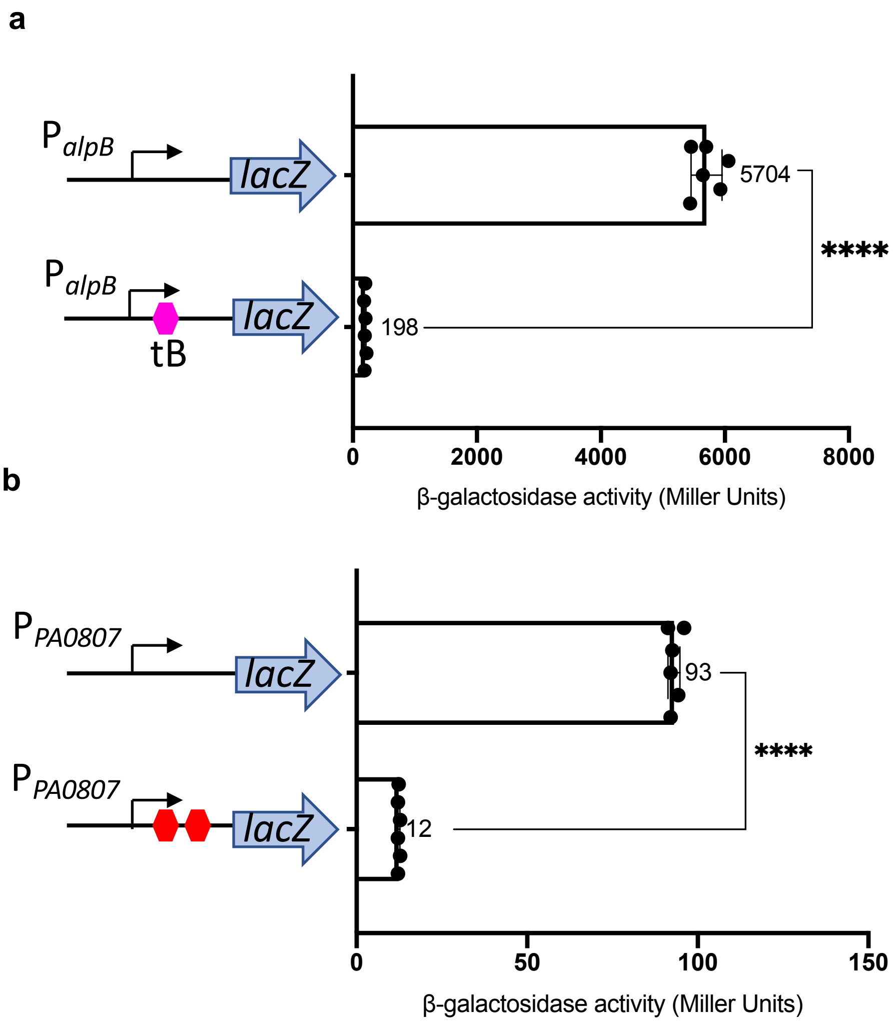

Supplementary Fig. 4. Intrinsic terminator located upstream of *alpB* ORF and downstream of *alpB* promoter. a Removal of the predicted terminator tB (pink hexagon) results in an increase in expression of the P*alpB-lacZ* reporter in cells that do not produce AlpA. β-galactosidase activity (in Miller Units) was measured from the indicated reporter constructs in *E. coli*. **b** Removal of the predicted intrinsic terminators (shown as red hexagons) downstream of the *PA0807* promoter results in increased expression of *lacZ* from the *PA0807* promoter in the absence of AlpA. β-galactosidase activity (in Miller Units) was measured from the indicated reporter constructs in PAO1. **a**,**b** Values and error bars reflect mean ± SD of n=3 biological replicates in technical duplicate. 2-tailed, unpaired, unequal variance t-tests were used to calculate p-values between indicated samples. p-values indicated by the following symbols: ≤0.0001 = ****.

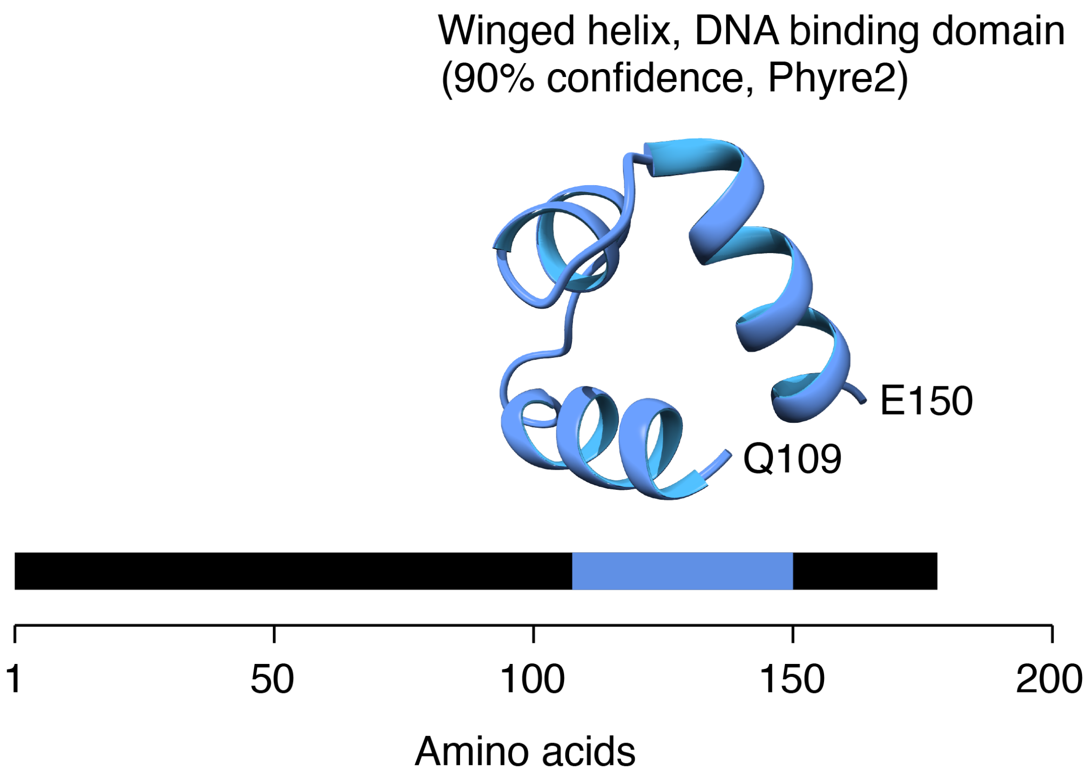

Supplementary Fig. 5. Predicted structure of putative winged helix-turn-helix DNA binding motif in AlpA. Phyre2[1] prediction of AlpA structure reveals a potential winged helix DNA binding domain from residues 109-150 in AlpA.

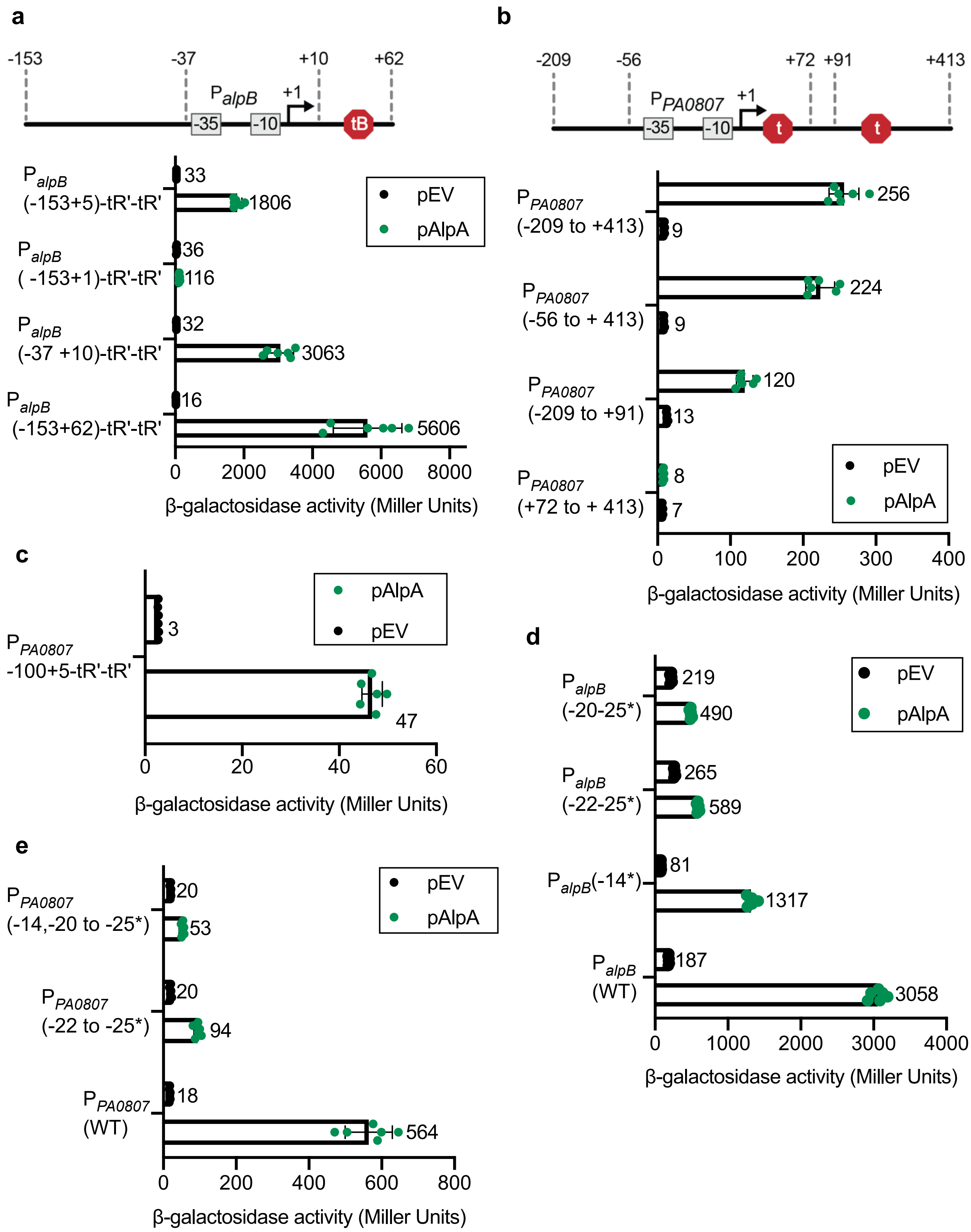

Supplementary Fig. 6. Identifying the minimal regions of the *alpB* and *PA0807* promoters that are responsive to AlpA. Diagram of *alpB* and *PA0807* promoter regions with red

hexagons representing predicted intrinsic terminators (**a**,**b**). β-galactosidase activity (in Miller Units) was measured from the indicated reporter constructs in *E. coli* (**a**,**c**) or PAO1 (**b**,**d**,**e**). Cells contained plasmid pAlpA (green) or plasmid pEV (the empty vector; black). Values and error bars reflect mean ± SD of n=3 biological replicates in technical duplicate. **a** *alpB* promoter reporter constructs of indicated lengths relative to TSS and containing tandem tR' terminators. **b** *PA0807* promoter reporter constructs of indicated lengths relative to TSS. **c** *PA0807* promoter reporter construct containing region -100 to +5 relative to TSS followed by two tandem tR' terminators upstream of the *lacZ* gene. **d** P_{alpB}-*lacZ* reporter assays with constructs harboring the indicated point mutations in the *alpB* promoter. A portion of the data in **d** are presented in Fig. 3b. **e** P_{PA0807}-*lacZ* reporter assays with constructs harboring the indicated point mutations in the *PA0807* promoter. **d**,**e** Numbers* in parentheses indicate nucleotides that were mutated in indicated promoter relative to predicted TSS.

a

b

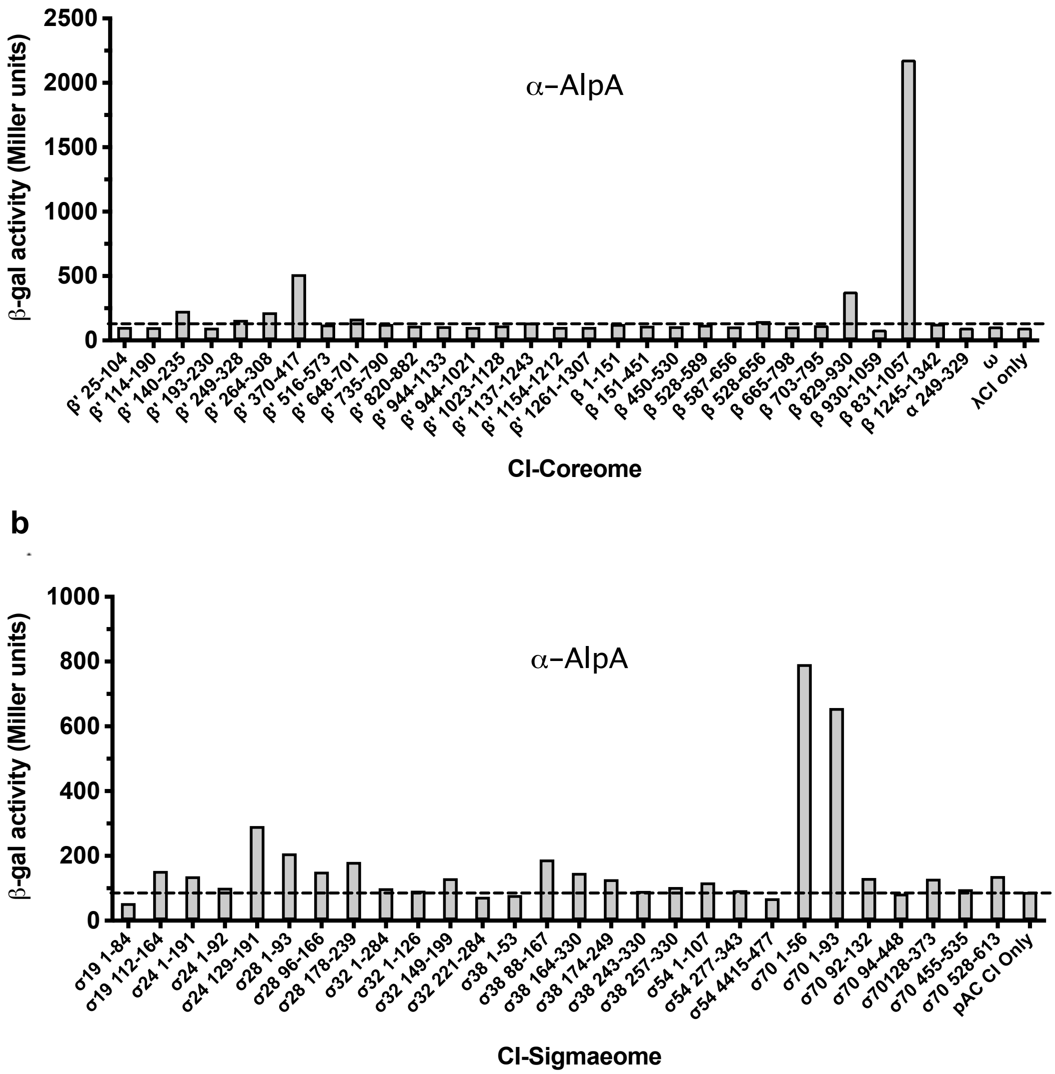

Supplementary Fig. 7. Bacterial 2-hybrid screen for interactions between AlpA and *E. coli* RNAP and sigma factors. β-galactosidase activity of *E. coli* reporter strain cells grown in the presence of 20 μM IPTG to induce synthesis of the indicated fusion proteins. **a** Library of surface exposed regions of *E. coli* RNAP core fused to λCI protein tested for interaction with AlpA fused to α-NTD. **b** Library of regions of different σ factors from *E. coli* fused to λCI protein tested for interaction with AlpA fused to α-NTD.

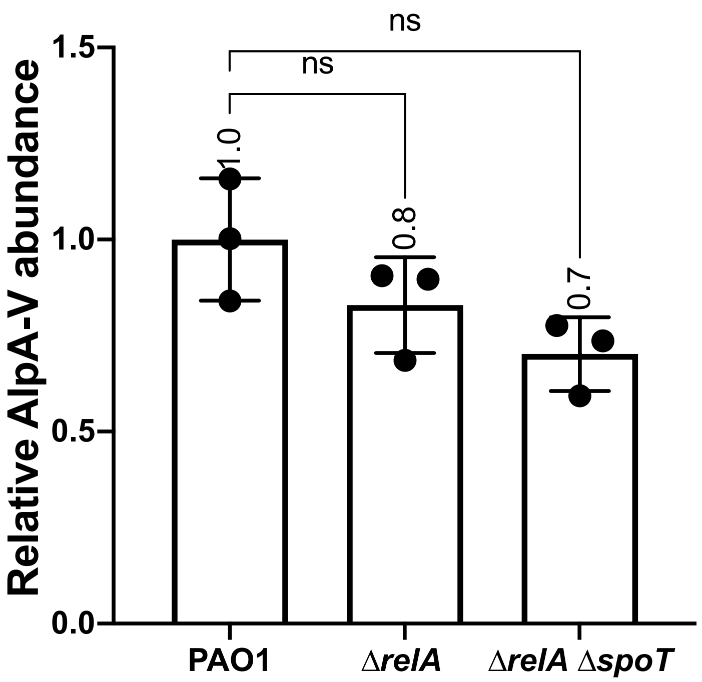

Supplementary Fig. 8. Western blot of AlpA abundance in PAO1, PAO1 Δ*relA* and PAO1 Δ*relA* Δ*spoT*. Quantification of relative AlpA-V abundance from biological triplicate samples. *alpA-V* was ectopically expressed from a plasmid under the control of an IPTG inducible promoter in PAO1 WT cells (indicated PAO1), PAO1 Δ*relA* mutant cells (indicated Δ*relA*), and PAO1 Δ*relA* Δ*spoT* mutant cells (indicated Δ*relA* Δ*spoT*). Values and error bars reflect mean ± SD of n=3 biological replicates. 2-tailed, unpaired, unequal variance t-tests were used to calculate p-values between indicated samples. p-values indicated by the following symbols: >0.05 =ns.

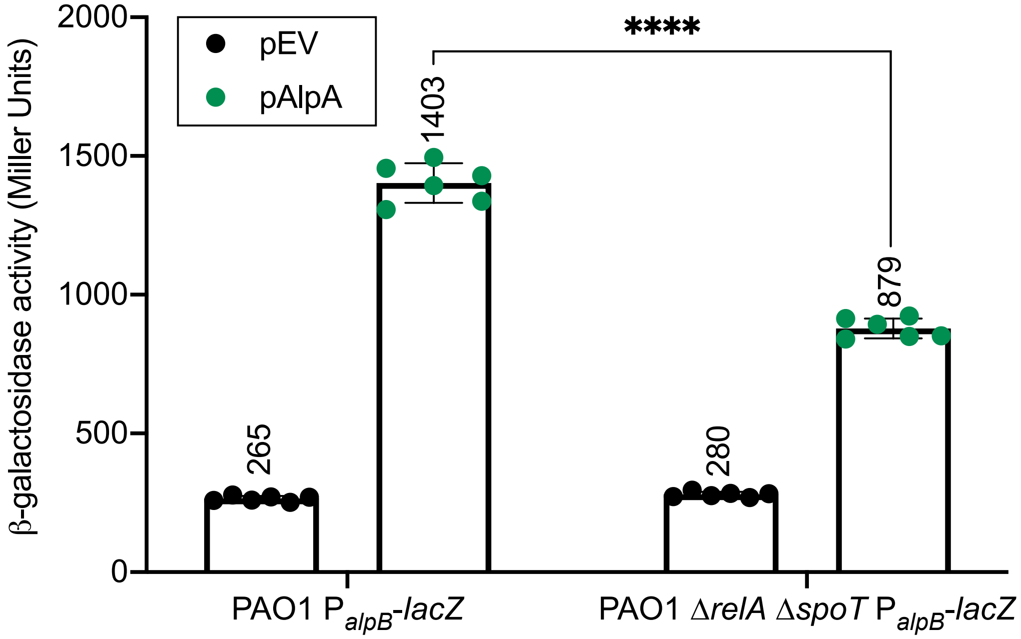

Supplementary Fig. 9. Decreased expression from AlpA regulated reporters in absence of ppGpp. β-galactosidase activity (in Miller Units) of an *alpB* promoter-*lacZ* reporter construct in *P. aeruginosa*. Strains were used that produce ppGpp (PAO1) or that cannot make ppGpp (PAO1 Δ*relA* Δ*spoT*). Cells contained plasmid pAlpA (green) or plasmid pEV (the empty vector; black). Graph shows a 2-fold reduction in *lacZ* expression from the *alpB* promoter in *P. aeruginosa* in Δ*relA* Δ*spoT* mutant cells compared to WT PAO1. Values and error bars reflect mean ± SD of n=3 biological replicates in technical duplicate. 2-tailed, unpaired, unequal variance t-tests were used to calculate p-values between indicated samples. p-values indicated by the following symbols: ≤0.0001 = ****.

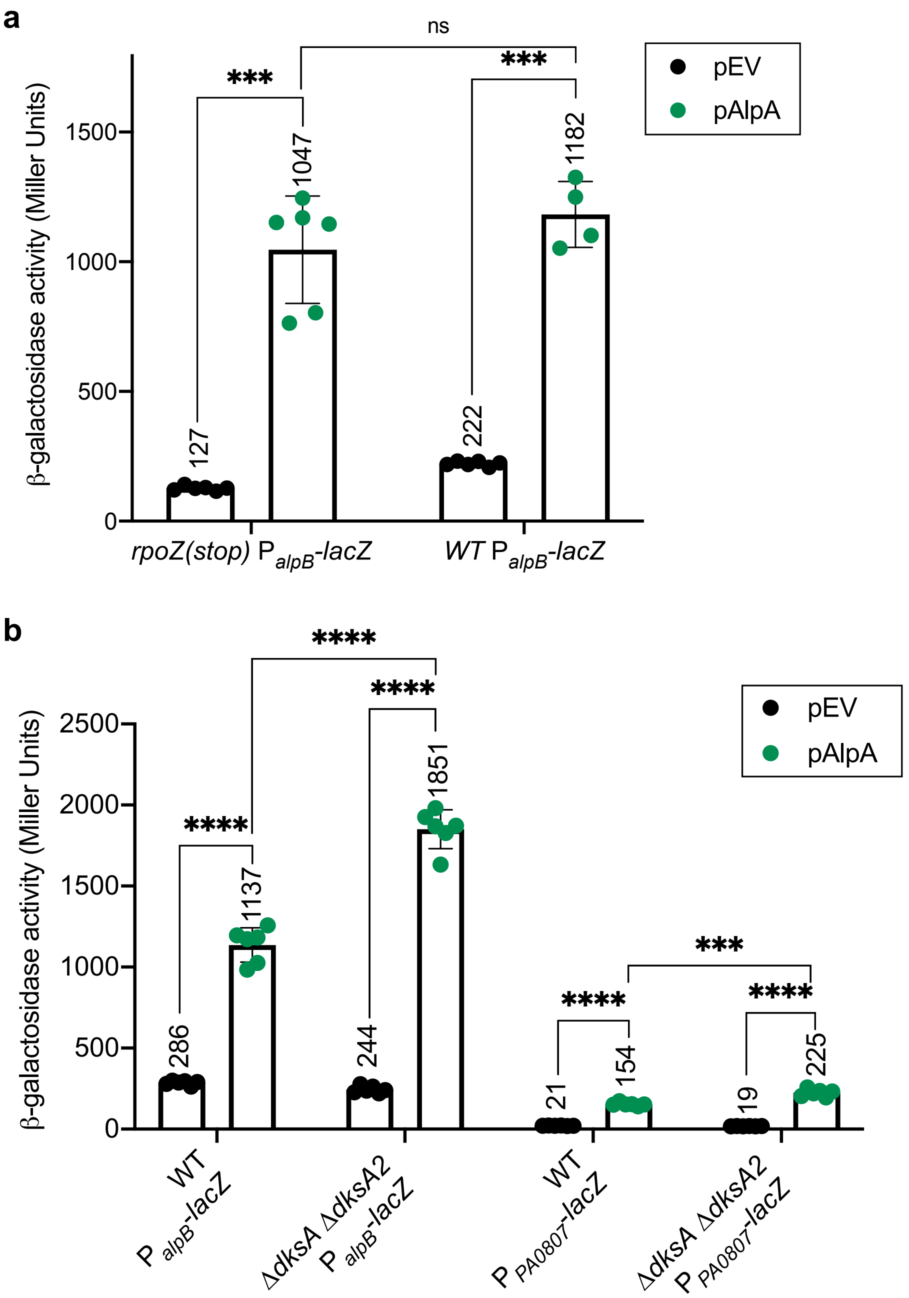

Supplementary Fig. 10. ppGpp binding sites 1 and 2 on *P. aeruginosa* RNAP do not appear to influence the expression of AlpA-regulated target genes. a β-galactosidase activity measured in Miller Units from the *alpB* promoter in cells that contain WT *rpoZ* (ω) or have a mutant *rpoZ* harboring a stop codon early on in the gene (*rpoZ*(stop)) and that contain an *alpA* expression plasmid (green) or empty vector (black). **b** Amount of β-galactosidase measured in Miller Units from *alpB* or *PA0807* promoter in cells that contain an *alpA* expression plasmid (grey) or empty vector (black) and that are WT or have *dksA* and *dksA2* deleted as indicated. Values and error bars reflect mean ± SD of n=3 biological replicates in technical duplicate. 2-tailed, unpaired, unequal variance t-tests were used to calculate p-values between indicated samples. p-values indicated by the following symbols: >0.05 =n.s., ≤0.001 = ***, ≤0.0001 = ****.

SUPPLEMENTARY TABLES

Supplementary Table 1. ChIP-Seq of AlpA-TAP. Table lists fold enrichment of specified regions of DNA following ChIP-Seq of AlpA-TAP supplied by a multicopy plasmid.

Peak Number	Fold Enrichment	Peak Maximum	Peak Start	Peak End	Peak Size	Closest Annotated Gene	Distance from translation start site*	Gene name	Gene product
1	9.74	884501	884071	896301	12230	PA0807	298	*ampDh3*	AmpDh3
2	4.92	1544284	1544109	1544422	313	PA1418	71	-	probable sodium:solute symport protein
3	3.58	4536671	4536400	4537010	610	PA4055	178	*ribC*	riboflavin synthase alpha chain
4	2.98	3507298	3507029	3507528	499	PA3125	47	-	hypothetical protein
5	2.81	3794829	3794342	3795651	1309	PA3391	-10	*nosR*	regulatory protein NosR
6	2.57	2114073	2113223	2114323	1100	PA1933	-887	-	probable hydroxylase large subunit

* Positive numbers indicate peak maximum is upstream of translation start; negative numbers indicate peak maximum is downstream of translation start

Supplementary Table 2. RNA-seq analysis of the effect of ectopically produced AlpA (from plasmid pAlpA) on expression of the indicated sense and antisense transcripts in PAO1 wild-type and PAO1 Δ*relA* mutant cells, as well as comparison of the abundance of the same transcripts in cells of the of Δ*relA* mutant and wild-type strain containing the empty vector (pEV). n=3 biological independent samples from cells of each strain. Differences in gene expression were assessed using DESeq2. The statistical significance of changes in gene expression was determined using the Wald test and adjusted for multiple testing using Benjamini-Hochberg false discovery rate correction. Fold changes in gene expression together with adjusted P-values are shown. In support of Fig. 5b and Fig. 5c.

Locus	Transcript	Fold change (PAO1 pAlpA vs PAO1 pEV)	Adjusted P-value	Fold change (PAO1 Δ*relA* pAlpA vs PAO1 Δ*relA* pEV)	Adjusted P-value	Fold change (PAO1 Δ*relA* EV vs PAO1 EV)	Adjusted P-value
PA1414 to *AS-PA1419*	*PA1414*	0.49	2.47E-02	0.91	0.841	0.48	0.014
	AS-PA1415	2.22	2.13E-07	1.32	0.133	1.60	0.003
	AS-PA1416	11.39	6.49E-17	4.79	2.62E-10	1.18	0.452
	AS-PA1417	29.45	2.97E-14	18.89	6.27E-16	1.01	0.939
	AS-PA1418	20.82	2.31E-15	8.88	5.67E-16	1.23	0.340
	AS-PA1419	18.77	4.89E-08	6.59	5.54E-06	1.07	0.662
PA5274 to *AS-PA5270*	*PA5274 (rnk)*	1.35	0.165	1.13	0.716	0.82	0.298
	AS-PA5273	6.19	1.04E-10	4.72	1.60E-07	0.82	0.366
	AS-PA5272	7.89	4.26E-24	3.25	5.03E-09	0.85	0.930
	AS-PA5271	9.71	2.77E-07	2.73	1.35E-02	1.03	0.889
	AS-PA5270	4.23	1.25E-17	2.25	8.23E-06	0.76	0.157

Supplementary Table 3. Strains used in this study.

Bacterial Strain	Source or Reference
PAO1 wild-type *P. aeruginosa*	Arne Rietsch, Case Western University
FW102 F'::placOL2-62-LacZ (aka BN469)	Bryce Nickles
SM10 (λpir)	Arne Rietsch, Case Western University
FW102	2
CSH100	2
DH5α F'IQ	Thermo Fisher
PAO1 Δ*alpB*	3
PAO1 Δ*alpBCDE* Δ*PA0807-PA0831*	This Study
PAO1 Δ*alpBCDE*	3
PAO1 Δ*alpA*	3
PAO1 Δ*relA* Δ*spoT*	This Study
PAO1 *alpA*(Stop)	3
PAO1 attB::CTX-*PalpB*(-153to+117)-lacZ	This Study
PAO1 attB::CTX-*PalpB*(-10 mutation)-lacZ	This Study
PAO1 attB::CTX-*PalpB*(-35 mutation)-lacZ	This Study
PAO1 attB::CTX-*PA0807*(-209 to +413)-lacZ	This Study
PAO1 attB::CTX-*PA0807*(-56 to + 413)-lacZ	This Study
PAO1 attB::CTX-*PA0807*(-209 to +91)-lacZ	This Study
FW102 F'::PlacUV5-tR'-tR'-lacZ	This Study
FW102 F'::*PalpB*(-153 to +62)-tR'-tR'-lacZ	This Study
PAO1 attB::CTX-*PalpB*(-153 to +117, mutation -25 to -22)-LacZ	This Study
FW102 F'::lacO-*PalpB*(153 to +6)-lacZ<br>FW102 F'::LacO-P*alpB*(-153 to +117)-*lacZ*	This Study<br>This Study
FW102 F'::P*lac*UV5-*lacZ*	This Study
FW102 F'::*PalpB*(-153 to +117)-tR'-tR'-*lacZ*	This Study
PAO1 P*PA0807*(-25 to -22 mutant)	This Study
PAO1 attB::CTX-P*alpB*(-153 to +117, -25 to -20 mutant)-*lacZ*	This Study
PAO1 attB::CTX-P*alpB*(-153 to +117, -14 mutant)-*lacZ*	This Study
PAO1 attB::CTX-PPA0807(-37 to +10)-lacZ	This Study
PAO1 attB::CTX-PPA0807(-37 to +413)	This Study
FW102 Δ*relA spoT*::cat	This Study
FW102 F'::P*alpB*(-37to+8)-tR'-tR'-*lacZ*	This Study

FW102 Δ*relA spoT*::cat F'::P*alpB*(-153 to +62)-tR'-tR'-lacZ	This Study
PAO1 attB::CTX-*PA0807*(-14,-20 to -25 mutant)-LacZ	This Study
PAO1 Δ*dksA* Δ*dksA2* attB::CTX-PalpB(-153 to 117)-LacZ	This Study
PAO1 Δ*dksA* Δ*dksA2* attB::CTX-*PA0807*(-209 to +91)-LacZ	This Study
FW102 F'::P*alpB*(-153 to +117)-tB-LacZ	This Study
PAO1 P*PA0807*(-14, -20 to -25 mutant)(ABE7)	This Study
PAO1 *rpoZ*(Stop) attB::CTX-P*alpB*(-153 to +117)-LacZ	This Study
PAO1 *rpoZ*(Stop) attB::CTX-*PA0807*(-209 to +91)-LacZ	This Study
PAO1 P*alpB*(-20 to -25 mutant)ABE6	This Study
PAO1 *alpA*-TAP	This Study
FW102 ΔrelA	4
FW102 Δ*relA spoT*::cat	This Study
FW102 Δ*relA spoT*::cat F'::PlacUV5-LacZ	This Study
FW102 F'::P*alpB*(-153+1)-tR'-tR'-LacZ	This Study
PAO1 Δ*alpB* Δ*relA*	This Study
PAO1 attB::CTX:P*PA0807*(-10 element mutant)-LacZ	This Study
PAO1 attB::CTX-P*PA0807* (-35 element mutant)-LacZ	This Study
PAO1 Tn7::GFP P*alpB*ABE6	This Study
PAO1 Tn7::mCherry	3

Supplementary Table 4. Plasmids used in this study.

Name	Plasmid	Source or Reference
	pFW11	2
	pPSV38	5
	pPSV38-*alpA*	3
	pPSV38-HSPK-AlpR-CTD	3
	pEXG2	6
	pMini-CTX-lacZ	7
	pAlpR-CTD	3
pJP01	pMini-CTX-P*alpB* (-153 to +117)-lacZ	This study
pJP06	pMini-CTX-P*alpB*(-10 element mutant, TATAAA → TcTAAc)-lacZ	This study
pJP07	pMini-CTX-P*alpB*(-35 element mutant, TTGACG → ccGAtG)-lacZ	This study
pJP09	pMini-CTX-*PPA0807*(-209 to +413)-lacZ	This study
pJP13	pMini-CTX-*PPA0807*(+72 to + 413)-lacZ	This study
pJP14	pMini-CTX-*PPA0807*(-56 to +413)-lacZ	This study
pJP15	pMini-CTX-*PPA0807*(-209 to +91)-lacZ	This study
pJP17	pMini-CTX-P*alpB*(-153 to +117, mutation in-25-22)-lacZ	This study
pJP24	pMini-CTX-P*PA0807*(-209 to +413, mutation in-25-22)-lacZ	This study
pJP38	pMini-CTX-P*alpB*(-153 to +117,mutations in -25 to -21)-lacZ	This study
pJP39	pMini-CTX-P*alpB*(-153 to +117 with point mutation at -14 G to T)-lacZ	This study
pJP44	pMiniCTX-Pbad-AlpA-VSV-G	This study
pJP154	pMini-CTX-P*PA0807*(-37 to +10)	This study
pJP155	pMini-CTX-P*PA0807*(-37 to +413)	This study
pJS10	PlacUV5-AlpA-VSV-G	This study
pJP50	pMini-CTX-P*alpB*(-153 to +117)-tB-lacZ	This study
pJP55	pMini-CTX-P*PA0807*(-209 to +413, mutations -14,-20to-25)-lacZ	This study
pJP135	pMini-CTX-PPA0807(-10 element mutant, TAGTTT→ cAGccT)-lacZ	This study

pJP136	pMini-CTX-*PPA0807* (-35 element mutant, TGCACG → ccCAtG)-lacZ	This study
pJP20	pFW11-PlacUV5-tR'-tR'-lacZ	This study
pJP19	pFW11-P*alpB*(-153 to +62)-lacZ	This study
pJP26	pFW11-LacO-P*alpB*(-153 to +117)-lacZ	This study
pJP27	pFW11-LacO-P*alpB*(-153 to +6)-lacZ	This study
pJP30	pFW11-P*lacUV5*-lacZ	This study
pJP32	pFW11-P*alpB*(-153 to +117)-tR'-tR'-lacZ	This study
pJP42	pFW11-P*alpB*(-37- to +8)-tR'-tR'-lacZ	This study
pJP51	pFW11-P*alpB* (-153 to +117)-lacZ	This study
pJP52	pFW11-P*alpB* ABE6-lacZ	This study
pJP53	pFW11-P*PA0807*(-209 to +413)-lacZ	This study
pJP54	pFW11-P*PA0807*(-209 to +413, -22 to -25 mutant)-lacZ	This study
pJP57	pFW11-P*PA0807* ABE7-lacZ	This study
pJP150	pFW11-P*alpB*(-117 to +1)-tR'-tR'-lacZ	This study
pJP151	pFW11-P*alpB*(-117 to +5)-tR'-tR'-lacZ	This study
pJP145	pEXG2-PalpB ABE6	This study
pJP59	pEXG2-P*PA0807* ABE7	This study
pJP137	pEXG2-*rpoZ*(Stop)	This study
pJP141	pEXG2-*alpA*-TAP	This study
pKMC1091	pEXG2-Δ*relA*	This study
pKMC1100	pEXG2-Δ*spoT*	This study
pKMC1073	pPSV37-*alpA*-TAP	This study
pJP48	pEXG2- Δ*dksA*	This study
pJP49	pEXG2-Δ*dksA2*	This study
	pBRα	8
	pACλCI32	9
	pACλCI-Ec βflap (831-1057)	10
pJS11	pACλCI-Paβflap(836-1074)	This study
pJP56	pBRα-AlpA	This study

pJS01	pACλCI -Pa σ70 (1-58)	This study
pJS02	pACλCI- Pa σ70 (1-95)	This study
pJP154	pKD3-spoT	This study
	pKD3	11
	pKD46	11
	pACλCI-β (1-151)	12
	pACλCI-β (151-451)	12
	pACλCI-β (450-530)	12
	pACλCI-β (528-589)	12
	pACλCI-β (587-656)	12
	pACλCI-β (528-656)	12
	pACλCI-β (665-798)	12
	pACλCI-β (703-795)	12
	pACλCI-β (829-930)	12
	pACλCI-β (930-1059)	12
	pACλCI-β (1254-1342)	12
	pACλCI-α (249-329)	12
	pACλCI-ω	12
	pACλCI-β' (25-104)	12
	pACλCI-β(114-190)	12
	pACλCI-β(140-235)	12
	pACλCI-β(193-230)	12
	pACλCI-β'(249-328)	12
	pACλCI-β'(264-308)	12
	pACλCI-β'(370-417)	12
	pACλCI-β'(516-573)	12
	pACλCI-β'(648-701)	12
	pACλCI-β'(735-790)	12
	pACλCI-β'(820-882)	12
	pACλCI-β'(944-1133)	12
	pACλCI-β'(1023-1128)	12
	pACλCI-β'(1137-1243)	12
	pACλCI-β'(1154-1212)	12
	pACλCI-β'(1261-1307)	12
	pACλCI-σ19(1-84)	This study
	pACλCI-σ19(112-164)	This study
	pACλCI-σ24(1-191)	This study

	pACλCI-σ24(1-92)	This study
	pACλCI-σ28(1-93)	This study
	pACλCI-σ24(129-191)	This study
	pACλCI-σ28(96-166)	This study
	pACλCI-σ28(178-239)	This study
	pACλCI-σ32(1-284)	This study
	pACλCI-σ32(1-126)	This study
	pACλCI-σ32(149-199)	This study
	pACλCI-σ32(221-284)	This study
	pACλCI-σ38(1-53)	This study
	pACλCI-σ38(88-167)	This study
	pACλCI-σ38(164-330)	This study
	pACλCI-σ38(174-249)	This study
	pACλCI-σ38(243-330)	This study
	pACλCI-σ38(257-330)	This study
	pACλCI-σ54(1-107)	This study
	pACλCI-σ54(277-343)	This study
	pACλCI-σ54(415-477)	This study
	pACλCI-σ70(1-56)	This study
	pACλCI-σ70(1-93)	This study
	pACλCI-σ70(92-132)	This study
	pACλCI-σ70(94-448)	This study
	pACλCI-σ70(128-373)	This study
	pACλCI-σ70(455-535)	This study
	pACλCI-σ70(528-613)	This study

Supplementary Table 5. Primers used in this study.

Primer name	Primer sequence 5' to 3'
PalpB-153_F	ATAgaattcCGAGCGGGTCGTCGCTTTGCTG
PalpB+117_R	ATAggatccCGCAGAGCTTGGCTCGGACATG
PalpB-10 mutation_F	GTGGAGAAGTTAGACCTTTATCGT
PalpB-10 mutation_R	ACGATAAAGGTCTAACTTCTCCAC
PalpB-35 mutation_F	TCCCATAGCATCGGTATGGGAAAA
PalpB-35 mutation_R	TTTTCCCATACCGATGCTATGGGA
PPA0807-209_F	ATAgaattcCAGGCGAAAAAAAGCCACTGTGC
PPA0807+413_R	ATAggatccGTTTTCACCTTTTTGCGGATGATG
PPA0807+72_F	ATAgaattcGCAACCGGACGCAGCGGGCG
PPA0807-56_F	ATAgaattcCGGCCAACGGACGAACGGTGTTGC
PPA0807+91_R	ATAggatccCGCCCGCTGCGTCCGGTTGC
PalpB-25to-22 mutation_F	CTATccctCGATAAAGGTATAAATTCTCC
PalpB-25to-22 mutation_R	TATCGagggATAGCGTCAATATGGGAAAA
PPA0807-25to-22 mutation_F	CACGCGGCccctCGCTCGCGGTAGTTTTTTCCCA
PPA0807-25to-22 mutation_R	ACCGCGAGCGagggGCCGCGTGCAACACCGTTC
PalpB-25to-20 mutation_F	TccctTTATAAAGGTATAAATTCTCC
PalpB-25to-20 mutation_R	CTTTATAAagggATAGCGTCAATATG
PalpB-14 mutation_F	GATAAAGTTATAAATTCTCCAC
PalpB-14 mutation_R	TTATAACTTTATCGTCCCATAG
AlpA-VSVG_R	TATggtaccTCATTTTCCTAATCTATTCATTTCAATATCTGT ATAGCGCTCGCCCACGATCCCCAC
araC-Ssp1_F	ATAaatattcaccgcggtggcggccGG
Pbad-Kpn1_R	ATAccatggGGTATTCCCTCCTTGGCCGGCCC
PPA0807-14-20-25 mutation_F	CACGCGGCtttcttCTCGCtGTAGTTTTTTCCCATG
PPA0807-14-20-25 mutation_R	GAAAAAACTACaGCGAGaagaaaGCCGCGTGCAACAC
PPA0807-10 mutation_R	GGCGACCACGGGAAACGTGATCATGGGAAAgAACgACC GCGA
PPA0807-10 mutation_F	GCGGCGGGACGCTCGCGGTcGTTcTTTCCCA
PPA0807-35 mutation_R	GAACGGTGTccCAtGCGGCGGGACGCTCG
PPA0807-35 mutation_R	GTCCCGCCGCaTGggACACCGTTCGTCCGTTG
PlacUV5_F	AAGAATTCAGGCTTTACACT
PalpB+62-Sal1_R	ATAgtcgacAGGCGGGCCCGGAACGGCCG
lacO-PalpB_F	GAAAACGGGTTAATTGTGAGCGGATAACAATTTTCCCAT ATTGACGCTATG
acO-PalpB_R	CAATATGGGAAAATTGTTATCCGCTCACAATTAACCCGT TTTCATGCAGAA
PalpB+43_R	gGATCCAATCGTGGAGAATTTATACCTTT
0908_reg_R_sal1	ATAgtcgacCGCAGAGCTTGGCTCGGACATG
PalpB-37_F	ATAgaattcTATTGACGCTATGGGACGATAAAG

PalpB_+10_R	ATAggatccTTTCAATCGTGGAGAATTTATAC
PalpB+8_10_mutF	CACGATTGcccTTCCTCTGGGTCGAC
PalpB+8_10_mutR	CCAGAGGAAgggCAATCGTGGAGAATTT
PalpB+117-HindIII_R	ATAaagcttCGCAGAGCTTGGCTCGGACATG
PPA0807+413-HindIII_R	ataaagcttGAGGTTCAGGGGCCGGCCAAG
PalpB+1_R	ATAGTCGACTGGAGAATTTATACCTTTATCGTCCCATAG CGTCAA
PalpB+5_R	ATAGTCGACATCGTGGAGAATTTATACCTTTATCGTCCC ATAGCGTC
sequencing del-0614-F	CACTGCGGTTGCAGCCGCAACAC
PAomega-stop-SOE_F	CGTTTCGAGgaattcTAATGAGCCACCAAGCGCGCCCGTC A
PA-omega-stop-SOE-R2	CTTGGTGGcTCATTAgaattcCTCGAAACGGTTATCGACGT TGTCCA
PAomeda-stop-3-Kpn1R	ataggtaccCGAGGTAGCTCGACAGTCTGTCGGCG
Paomega sequencing stop_R	GTAGTAGTAGGCGCGGCGGACCAGG
AlpA-TAP-Kpn1_R	ataggtaccCCGACGCCAGCCATTGCGCTGG
AlpA-TAP-BamH1_F	ataggatccAAAAATTTCTGCATGAAAACGGGTTTTCCCATA TTGACGCTATGG
del-DksA 5'_R	ATAggcgcgccCATGAATGGCCGCCTCTCACTTTC
del-DksA 5'_F	ATAggatccGATCGGCTCGGCCGTGGTGAAG
del-DksA 3'_R	ATAgaattcCTGCGGCGTAGGCCGGGAAGCGTTC
del-DksA 3'_F	ATAggcgcgcccTGAGCCGGACCGACGGGGCG
sequence del-DksA_F	CAGGCCGCGGGCGGCGGTGTAG
del-DksA2 5'_F	ATAaagcttcGACGCTGTAGTGGTTGCCCAG
del-DksA2 5'_R	ATAgcggccgcCATGGGCTGAACCTGGGAAATTTAAAACG
del-DksA2 3'_F	ATAgcggccgccTGAAGAGGTTACCAACCGTGAACCAAC
del-DksA2 3'_R	ATAgaattcCAGCTCGTCGCGGAAGGTGAAG
sequence del-DksA2_F	GTCCGCTCGCCGCGTCGTAGC
pKD3_spoT-del_F	cccttgtatctgtttgaaagcctgaatcaactgattcaaacctaccatatgaatatcct ccttagttcctattccgaag
pKD3_spoT_del_R	ggcgagcatttcgcagatgcgtgcataacgtgttgggttcataaagtgtaggctgg agctgcttcgaagttcctatactt
sequencing del-spoT_R	gaacttcatgtacgccaacggcatc
sequencing_cat_F	ctggcctatttccctaaagggttt
qPCR PalpB_ChIP_F	TTGCATTTCCGGGAGTCATC
qPCR PalpB_ChIP_R	AAGGCGTTGCCATCGATAC
int_miniCTX	CCCTTTAGTGAGGGTTAATT
pser_up	cgagtggtttaaggcaacggtcttga
qPCR_Ec_clpX_F	GGTCGCGGTATACAACCATTAC
qPCREc_clpX_R	GGACCGATCAGCAGAATGTTAC

qPCR-PAO1-clpX_F	GTCAGGAGATGGGCAAGAAG
qPCR-PAO1-clpX_R	GCCCTACAAACTCGGGAATAA
qPCR PA0910_F	GAGTCCTTGAGCCTGCTTG
qPCR PA0910_R	CCAGGTCAGGCGAAAGATG
qPCR PA0829_F	GCTATATCGCCTGGAACTTCG
qPCR PA0829_R	CACCTTGCGCATCTGGTAA
qPCR PA0807_F	ATGCATTTCCGCCCGGAGAACTA
qPCR PA080_R	CGGGTATTTCTCGTTCAGAGCGTA
qPCR PA0815_F	ATGATCGACCTGGTGATGCTCAAG
qPCR PA0815_R	ACTGTTCCAGGGTCTGGATACGC
PAsigma70-1-56-F	ATA GCGGCCGC C ATGTCCGGAAAAGCGCAACAGCA
PA-sigma70-1-56-R	ATA GGATCC TTA GAATACGTTGATCCCCATGTCGTTGAT
PAsigma70-1-95-R	ATA GGATCC TTA GCGACCGATGTCGCTTTCCACCGCGGCCA
Pa-B-Flap_Not1_F2	ata gcggccgc c ATCCACATCCAGGAACTGACCT
Pa_B-flap_BamH1-stop_R2	ataggatccttaCTTGATCGCCAGGTAGACCTTGA
VSVG-insert-F	TGGGGATCGTGGGCGAGCGCGCGGCCGCATATACAGATATT
VSVG-insert-R	CCGGGTACCGAGCTCGAATTCTCATTTTCCTAATCTATTCATTTCA
0807-37_EcoR1-F	atagaattcGTTGCACGCGGCGGGACG
0807-37+10-ultra-BamH1-R	gatccACGTGATCATGGGAAAAAACTACCGCGAGCGTCCCGCCGCGTGCAACg
0807-37+10-ultra-F	aattcGTTGCACGCGGCGGGACGCTCGCGGTAGTTTTTTCCCATGATCACGTg

SUPPLEMENTARY REFERENCES

1. Kelley, L. A. *et al*. The Phyre2 web portal for protein modeling, prediction and analysis. *Nature Protocols* **10**, 845-858 (2015).
2. Whipple, F. W. Genetic analysis of prokaryotic and eukaryotic DNA-binding proteins in *Escherichia coli*. *Nucleic Acids Res.* **26**, 3700-3706 (1998).
3. McFarland, K. A. *et al.* A self-lysis pathway that enhances the virulence of a pathogenic bacterium. *Proc. Natl. Acad. Sci. U S A* **112**, 8433–8438 (2015).
4. Rohlfing, A. E., Ramsey, K. M., & Dove, S. L. Polyphosphate kinase antagonizes virulence gene expression in *Francisella tularensis*. *J. Bacteriol*. **200**, e00460-17 (2018).
5. Goldman, S. R. *et al.* NanoRNAs Prime Transcription Initiation In Vivo. *Mol. Cell* **42**, 817–825 (2011).
6. Rietsch, A., Vallet-Gely, I., Dove, S. L. & Mekalanos, J. J. ExsE, a secreted regulator of type III secretion genes in *Pseudomonas aeruginosa*. *Proc Natl Acad Sci U S A* **102**, 8006–8011 (2005).
7. Hoang, T. T., Kutchma, A. J., Becher, A. & Schweizer, H. P. Integration-Proficient Plasmids for *Pseudomonas aeruginosa*: Site-Specific Integration and Use for Engineering of Reporter and Expression Strains. *Plasmid* **43**, 59–72 (2000).
8. Dove, S. L., Joung, J. K., & Hochschild, A. Activation of prokaryotic transcription through arbitrary protein-protein contacts. *Nature* **386**, 627-630 (1997).
9. Dove, S. L., & Hochschild, A. Conversion of the ω subunit of *Escherichia coli* RNA polymerase into a transcriptional activator or an activation target. *Genes Dev.* **12**, 745-754 (1998).
10. Deighan, P., Diez, C. M., Leibman, M., Hochschild, A. & Nickels, B. E. The bacteriophage lambda Q antiterminator protein contacts the beta-flap domain of RNA polymerase. *Proc. Natl. Acad. Sci.* **105**, 15305–15310 (2008).
11. Datsenko, K. A. & Wanner, B. L. One-step inactivation of chromosomal genes in *Escherichia coli* K-12 using PCR products. *Proc Natl Acad Sci U S A* **97**, 6640–6645 (2000).
12. Wang Erickson, A. F. *et al*. A novel RNA polymerase-binding protein that interacts with a sigma-factor docking site. *Mol. Microbiol*. **105**, 652-662 (2017).
