# Supplementary material for: Control of a programmed cell death pathway in Pseudomonas aeruginosa by an antiterminator
Source: Nat Commun. 2021 Mar 17;12:1702. doi: 10.1038/s41467-021-21941-7 (PMC7969949; doi:10.1038/s41467-021-21941-7)
Supplement: Supplementary file 3 — Description of Additional Supplementary Files [file 41467_2021_21941_MOESM3_ESM.pdf]

Description of Additional Supplementary Files

File Name: Supplementary Data 1
Description: RNA-Seq analysis of genes controlled by AlpA. Comparison of PAO1 Δ*alpA* mutant cells to PAO1 WT cells following treatment with ciprofloxacin. n=3 biological independent samples from cells of each strain. Differences in gene expression were assessed using DESeq2. The statistical significance of changes in gene expression was determined using the Wald test and adjusted for multiple testing using Benjamini-Hochberg false discovery rate correction. Mean expression level, log2 fold changes in gene expression and adjusted P-values are shown.

File Name: Supplementary Data 2
Description: RNA-Seq analysis of genes controlled by AlpA. Comparison of PAO1 *alpA*(stop) mutant cells to PAO1 WT cells following treatment with ciprofloxacin. n=3 biological independent samples from cells of each strain. Differences in gene expression were assessed using DESeq2. The statistical significance of changes in gene expression was determined using the Wald test and adjusted for multiple testing using Benjamini-Hochberg false discovery rate correction. Mean expression level, log2 fold changes in gene expression and adjusted P-values are shown.

File Name: Supplementary Data 3
Description: RNA-Seq analysis of the effects of ectopic *alpA* expression in PAO1 WT cells. RNA-seq was performed with PAO1 WT cells containing plasmid pAlpA or pEV (empty vector control plasmid). n=3 biological independent samples from cells of each strain. Differences in gene expression were assessed using DESeq2. The statistical significance of changes in gene expression was determined using the Wald test and adjusted for multiple testing using Benjamini-Hochberg false discovery rate correction. Mean expression level, log2 fold changes in gene expression and adjusted P-values are shown.

File Name: Supplementary Data 4
Description: RNA-Seq analysis of the effects of ectopic *alpA* expression in PAO1 Δ*relA* mutant cells. RNA-seq was performed with PAO1 Δ*relA* mutant cells containing plasmid pAlpA or pEV (empty vector control plasmid). n=3 biological independent samples from cells of each strain. Differences in gene expression were assessed using DESeq2. Mean expression level, log2 fold changes in gene expression and adjusted P-values are shown.

File Name: Supplementary Movie 1
Description: Time-lapse microscopy of PAO1 mCherry with a WT *alpB* promoter (synthesizing mCherry and shown in red) and PAO1 GFP P_{alpB}ABE6 (not synthesizing

mCherry, shown in phase contrast) ectopically synthesizing the AlpR-CTD. Experiment was performed independently twice with similar results. Images were acquired every 5 min for 6 hrs at 37 ˚C.
