# Supplementary material for: Control of a programmed cell death pathway in Pseudomonas aeruginosa by an antiterminator
Source: Nat Commun. 2021 Mar 17;12:1702. doi: 10.1038/s41467-021-21941-7 (PMC7969949; doi:10.1038/s41467-021-21941-7)
Supplement: Supplementary file 9 — Reporting Summary [file 41467_2021_21941_MOESM9_ESM.pdf]

nature research

Corresponding author(s): Simon L. Dove

Last updated by author(s): Feb 4, 2021

Reporting Summary

Nature Research wishes to improve the reproducibility of the work that we publish. This form provides structure for consistency and transparency in reporting. For further information on Nature Research policies, see our Editorial Policies and the Editorial Policy Checklist.

Statistics

For all statistical analyses, confirm that the following items are present in the figure legend, table legend, main text, or Methods section.

n/a	Confirmed	
☐	☒	The exact sample size (n) for each experimental group/condition, given as a discrete number and unit of measurement
☐	☒	A statement on whether measurements were taken from distinct samples or whether the same sample was measured repeatedly
☐	☒	The statistical test(s) used AND whether they are one- or two-sided<br>*Only common tests should be described solely by name; describe more complex techniques in the Methods section.*
☒	☐	A description of all covariates tested
☐	☒	A description of any assumptions or corrections, such as tests of normality and adjustment for multiple comparisons
☐	☒	A full description of the statistical parameters including central tendency (e.g. means) or other basic estimates (e.g. regression coefficient) AND variation (e.g. standard deviation) or associated estimates of uncertainty (e.g. confidence intervals)
☐	☒	For null hypothesis testing, the test statistic (e.g. F, t, r) with confidence intervals, effect sizes, degrees of freedom and P value noted<br>*Give P values as exact values whenever suitable.*
☒	☐	For Bayesian analysis, information on the choice of priors and Markov chain Monte Carlo settings
☒	☐	For hierarchical and complex designs, identification of the appropriate level for tests and full reporting of outcomes
☒	☐	Estimates of effect sizes (e.g. Cohen's d, Pearson's r), indicating how they were calculated

Our web collection on statistics for biologists contains articles on many of the points above.

Software and code

Policy information about availability of computer code

Data collection	qRT-PCR and qPCR data were collected using a LightCycler96 (Roche) with LightCycler software version 1.1 (Roche). Image acquisition software used for time lapse microscopy images was Nikon Elements 4.30 acquisition Software (Nikon).
Data analysis	For RNA-Seq analyses of samples treated with ciprofloxacin, reads were demuliplexed based on their associated barcode sequence using custom scripts [https://github.com/broadinstitute/split_merge_pl], aligned using BWA (version 0.7.15) and read counts were assigned to genes and other genomic features using custom scripts [https://github.com/broadinstitute/BactRNASeqCount]. Differential expression analysis was conducted with DESeq2. For RNA-Seq analyses of cells with ectopic AlpA, reads were aligned using bowtie2 v. 2.3.4.1, read counts were assigned to features using HTSeq-count version 0.11.1, and differential expression analysis was performed with DESeq2 version 1.20. For ChIP-Seq analyses, reads were aligned using bowtie 2-2.0.6. Regions of enrichment were called using QuEST, version 2.42.

For manuscripts utilizing custom algorithms or software that are central to the research but not yet described in published literature, software must be made available to editors and reviewers. We strongly encourage code deposition in a community repository (e.g. GitHub). See the Nature Research guidelines for submitting code & software for further information.

Data

Policy information about availability of data

All manuscripts must include a data availability statement. This statement should provide the following information, where applicable:

- Accession codes, unique identifiers, or web links for publicly available datasets
- A list of figures that have associated raw data
- A description of any restrictions on data availability

RNA-Seq and ChIP-Seq data that support the findings of this study have been deposited in the National Center for Biotechnology Information Gene Expression Omnibus with the accession code number GSE152485 and are available at: https://www.ncbi.nlm.nih.gov/geo/query/acc.cgi?acc=GSE152485

Field-specific reporting

Please select the one below that is the best fit for your research. If you are not sure, read the appropriate sections before making your selection.

- [x] Life sciences
- [ ] Behavioural & social sciences
- [ ] Ecological, evolutionary & environmental sciences

For a reference copy of the document with all sections, see nature.com/documents/nr-reporting-summary-flat.pdf

Life sciences study design

All studies must disclose on these points even when the disclosure is negative.

Sample size	No sample size calculation was performed. Biological triplicate samples were used in experiments as this is standard practice in the field.
Data exclusions	No data were excluded from the analyses.
Replication	Reproducibility of our experiments was accomplished by performing experiments with 3 biological replicates and reproducible data were observed. Gene expression experiments were performed a minimum of two times (each with 3 biological replicates), with reproducible results. Time-lapse microscopy experiments were performed twice with reproducible results. RNA-Seq and ChIP-Seq experiments were performed once with biological triplicate samples. RNA-Seq studies aimed at identifying those genes regulated by AlpA in response to DNA-damage were verified by comparing results obtained with cells lacking the alpA gene (Supplementary Data 1) and those obtained with cells that contained a stop codon in the alpA ORF (Supplementary Data 2). RNA-Seq studies were also verified independently by qRT-PCR analyses of the expression of select genes. Findings with ChIP-Seq studies were verified by independent ChIP-qPCR analyses.
Randomization	N/A
Blinding	Studies were not blinded during collection of the data. Blinding was not necessary as studies did not involve animal or human subjects.

Reporting for specific materials, systems and methods

We require information from authors about some types of materials, experimental systems and methods used in many studies. Here, indicate whether each material, system or method listed is relevant to your study. If you are not sure if a list item applies to your research, read the appropriate section before selecting a response.

Materials & experimental systems

n/a	Involved in the study	
[ ]	[x]	Antibodies
[x]	[ ]	Eukaryotic cell lines
[x]	[ ]	Palaeontology and archaeology
[x]	[ ]	Animals and other organisms
[x]	[ ]	Human research participants
[x]	[ ]	Clinical data
[x]	[ ]	Dual use research of concern

Methods

n/a	Involved in the study	
[ ]	[x]	ChIP-seq
[x]	[ ]	Flow cytometry
[x]	[ ]	MRI-based neuroimaging

Antibodies

Antibodies used	Human IgG coupled to a 6% agarose matrix (IgG Sepharose 6 Fast Flow, GE Healthcare, 17-0969-01). Anti VSV-Glycoprotein antibody (mouse monoclonal, clone P5D4, SigmaAldrich, Cat# SAB4200695). Purified anti-E.coli RNA polymerase alpha antibody, clone 4RA2 (BioLegend Cat# 663104). IRDye 680LT donkey anti-mouse IgG (LiCor 926-68022).
Validation	For the validation of the primary antibody against the alpha subunit of RNA polymerase the product data sheet indicates reactivity to be "E. coli, widely among Gram-negative bacteria, some Gram-positive bacteria". For validation of the primary antibody against the VSV-G epitope tag the product data sheet indicates that the antibody recognizes the 5 C-terminal amino acids of VSV-G and can be used in immunoblotting.

ChIP-seq

Data deposition

- [x] Confirm that both raw and final processed data have been deposited in a public database such as GEO.
- [x] Confirm that you have deposited or provided access to graph files (e.g. BED files) for the called peaks.

Data access links<br>*May remain private before publication.*	https://www.ncbi.nlm.nih.gov/geo/query/acc.cgi?acc=GSE152485
Files in database submission	Original ChIP-Seq read files: 0907_C-TAP_1.fastq, 0907_C-TAP_2.fastq, 0907_C-TAP_3.fastq, 0907_1.fastq, 0907_2.fastq, 0907_3.fastq<br>Peaks identified: GSE152481_0907_C-TAP_peaks.bed.gz.
Genome browser session<br>(e.g. UCSC)	No longer applicable.

Methodology

Replicates	Experiments were performed with biological triplicate samples.
Sequencing depth	Between approximately 4.5 – 7.1 million single-end 36 base pair reads were obtained for each biological replicate. Of these, 95.2 – 97.9% mapped to reference sequences (the PAO1 genome, NC_002516.2 and the expression plasmid, pAlpA-TAP). This corresponds to 25 – 40x coverage of the reference genome.
Antibodies	Human IgG coupled to a 6% agarose matrix (IgG Sepharose 6 Fast Flow, GE Healthcare, 17-0969-01).
Peak calling parameters	Reads were mapped to the PAO1 genome (NC_002516.2) and the expression plasmids pPSV37-AlpA-TAP and pPSV37-AlpA using bowtie2-2.0.6 allowing up to one mismatch per seed. The program QuEST (version 2.42) was used to call peaks. All the mock IP (PAO1 with the vector pPSV37-AlpA) replicate data was merged and used as “background” for each biological replicate (PAO1 with the vector pPSV37-AlpA-TAP). Peaks were called using the following parameters: KDE bandwidth = 30, Region = 300, Mappable genome fraction = 1, ChIP_enrichment_threshold = 1, ChIP_extension_enrichment = 1.5, ChIP_to_background_ratio = 2.
Data quality	Regions in each biological replicate were considered peaks if they are 2.5-fold enriched for reads over background, have a positive peak shift and strand correlation, and have a q-value of less than 0.01. AlpA peaks are defined as the minimal region identified in at least two biological replicates, resulting in 6 peaks ranging in enrichment from 2.6 – 9.7-fold enriched over background and one peak with greater than 5-fold enrichment.
Software	The program QuEST (version 2.42) was used to analyze ChIP-Seq data.
